# Breaking the Chains of Therapeutic Blockade: Pyroptosis-Induced Photothermal-Chemotherapy with Targeted Nanoprobes in Triple-Negative Breast Cancer

**DOI:** 10.34133/bmr.0200

**Published:** 2025-05-15

**Authors:** Zuying Li, Kexiao Yu, Youde Cao, Hui Yuan, Lingcheng Wu, Linyan Xiong, Yi Tang, Bing Liang

**Affiliations:** ^1^ Department of Ultrasound of Children’s Hospital of Chongqing Medical University, National Clinical Research Center for Child Health and Disorders, Ministry of Education Key Laboratory of Child Development and Disorders, Chongqing Engineering Research Center of Stem Cell Therapy, Children’s Hospital of Chongqing Medical University, Chongqing 400014, P. R. China.; ^2^ Department of Ultrasound & Chongqing Key Laboratory of Ultrasound Molecular Imaging, The Second Affiliated Hospital of Chongqing Medical University, Chongqing 400010, P. R. China.; ^3^ Department of Orthopedics, Chongqing Traditional Chinese Medicine Hospital, The First Affiliated Hospital of Chongqing University of Chinese Medicine, Chongqing 400021, P. R. China.; ^4^ Department of Pathology from College of Basic Medicine, and Molecular Medicine Diagnostic & Testing Center, and Department of Clinical Pathology Laboratory of Pathology Diagnostic Center, Chongqing Medical University, Chongqing 400016, P. R. China.; ^5^ Department of Pathology, The First Affiliated Hospital of Chongqing Medical University, Chongqing, 400042, P. R. China.

## Abstract

There is an important clinical need and social significance, especially for young patients, to explore a new breast-conserving strategy that is not dependent on biomarkers for anti-triple-negative breast cancer. Disulfiram, historically employed for the treatment of chronic alcoholism, has recently emerged as a promising antitumor agent in combination with Cu^2+^. However, reported disulfiram–Cu^2+^ codelivery regimens often suffer from instability as well as inadequate drug metabolism, which is detrimental to the production and action of the antitumor active ingredient copper(II) bis(diethyldithiocarbamate). To address this obstacle, this study tested nanosystems ICG-CuET@PLGA-CS-HA (IC@PCH) nanoparticles (NPs) carrying the chemotherapeutic agent copper(II) bis(diethyldithiocarbamate) and photosensitizer indocyanine green for the efficient delivery of antitumor drugs. Benefiting from the involvement of hyaluronic acid, the prepared IC@PCH NPs not only targeted CD44 on the surface of tumor cells but also showed a longer in vivo circulation time. The in vitro and in vivo results demonstrated that IC@PCH NP-mediated photothermal-chemotherapy treatment led to pyroptosis via the NLRP3/caspase-1 classical pathway, which had a significant therapeutic effect on triple-negative breast cancer. In addition, targeting IC@PCH NPs allows photoacoustic–magnetic resonance–fluorescence trimodal imaging, which is capable of detecting more insidious cancer foci and opens up new avenues for precise cancer diagnosis and treatment.

## Introduction

The latest World Health Organization statistics show that breast cancer (BC) has surpassed lung cancer globally for both incidence and mortality rates in the female population [1]. Actors like genetics, environment, and lifestyle are driving BC prevalence up annually and at younger ages [2,3]. Triple-negative breast cancer (TNBC) is a subtype of BC that lacks the expression of the estrogen receptor, the progesterone receptor, and human epidermal growth factor receptor 2 (HER2). Due to the lack of therapeutic targets, TNBC is insensitive to endocrine therapy as well as anti-HER2 targeted therapy treatment, and the prognosis is often poor [4,5]. This necessitates an emphasis on the diagnosis and treatment of middle-aged and young female TNBC patients. Current treatments involve surgery, radiation, chemotherapy, and neoadjuvant chemotherapy [6,7]. However, these typically lack specificity, causing emotional trauma of surgery and severe adverse effects from radiotherapy/chemotherapy [8,9]. Thus, to explore a new breast-conserving strategy, without being dependent on biomarkers, anti-TNBC has a marked clinical need and social significance for young patients.

Disulfiram (DSF) has been approved by the Food and Drug Administration for the treatment of alcohol dependence since 1951 and was first found to have anticancer effects in 1977 [10]. Since then, several studies have demonstrated that DSF has excellent tumor toxicity, with this efficacy being strongly contingent upon the presence of Cu^2+^ [11]. In vivo, DSF is metabolized to dithiocarbamate, and its antitumor activity is greatly enhanced after liganding with Cu^2+^ to form a dithiocarbamate–copper complex (copper(II) bis(diethyldithiocarbamate) [CuET]), considering the determining metabolite for the tumor-suppressing effect [12,13]. Several studies have demonstrated that DSF has excellent tumor toxicity and plays an important role in mitigating drug resistance, inhibiting angiogenesis, suppressing tumor metastasis, and modulating the immune microenvironment [14,15]. Despite the proven efficacy of DSF combined with Cu^2+^, its in vivo transportation faces many challenges in practical use. Clinical trials of oral as well as intravenous DSF/Cu^2+^ supplements have shown disappointing results due to the fact that DSF is highly unstable under the biological conditions of gastric acid and blood [16]. What is more, to make matters worse, single treatments are often shown to be less effective [17]. It is essential to probe into other effective and efficient strategies against TNBC.

Nanomedicine is a new field of medicine that utilizes nanomaterials for disease diagnosis and treatment at the molecular level, with little invasiveness and side effects [18–21]. Using nanodrug delivery systems to deliver DSF-based drugs is an exciting choice. However, DSF/Cu^2+^ codelivery nanosystems cannot ensure the efficient generation of the antitumor active ingredient CuET in vivo. This is due to the inconsistent pharmacokinetic profiles of Cu^2+^ and DSF, and the formation of CuET in tumors is not efficient and selective, greatly impairing therapeutic efficacy [22]. Meanwhile, most DSF-based delivery nanosystems are passively targeted to reach the tumor site just through the enhanced permeability and retention effect, which cannot achieve tumor site-specific accumulation ideally [23,24]. To overcome the above difficulties, we developed a nanoparticle (NP)-based targeted delivery system to increase the stability of CuET during systemic circulation by poly(lactic-*co*-glycolic acid) (PLGA) enclosure wrapped CuET. Based on previous studies, hyaluronic acid (HA) was loaded around the outermost layer of NPs, which specifically targeted CD44-overexpressing TNBC cells, without the systemic toxicity and solubility issues of chemotherapeutic drugs [25,26]. After precise targeting, combination therapy is carried out at the same time, which is expected to be anti-TNBC.

**Fig. 1.**
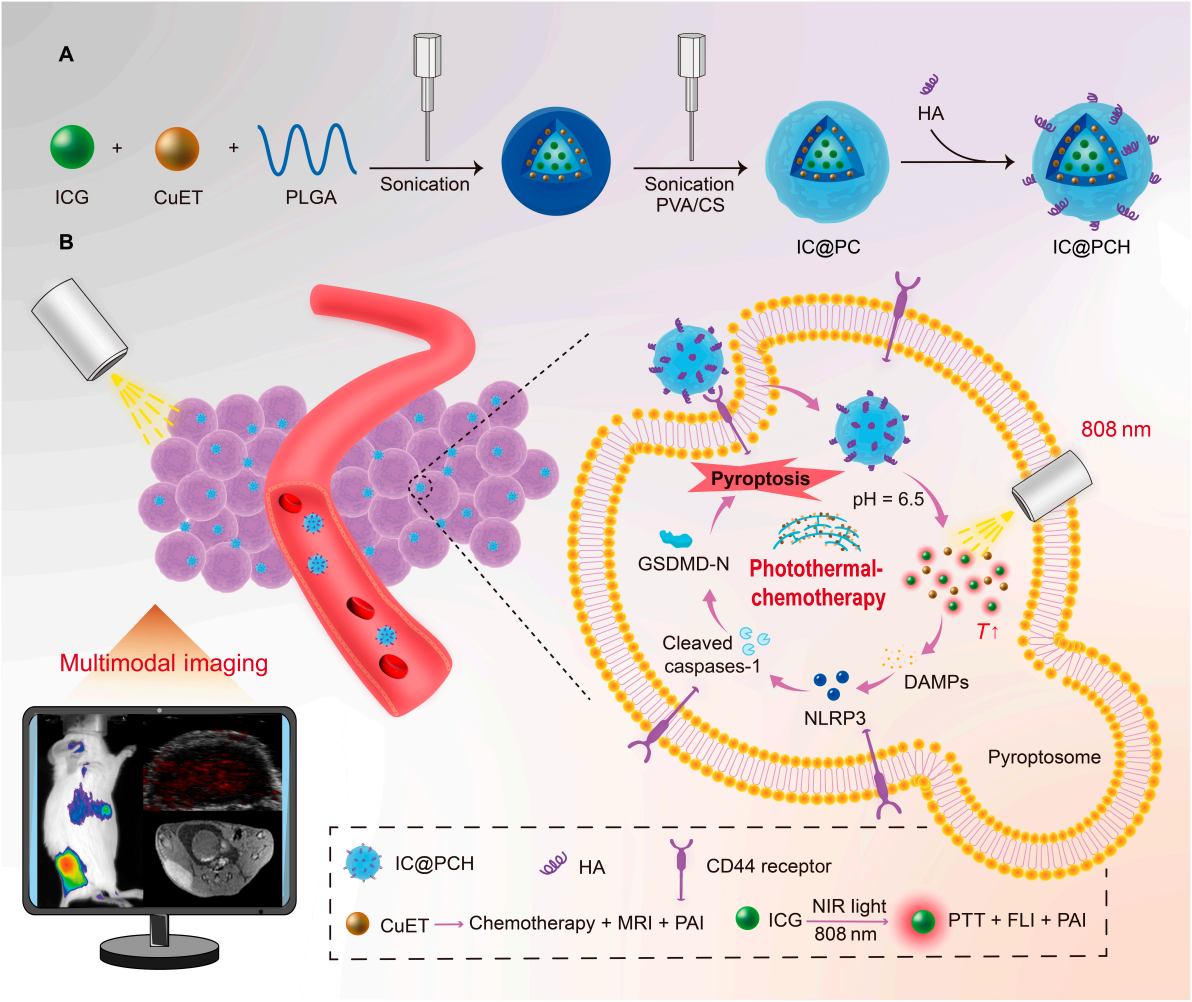
(A) Diagram of the ICG-CuET@PLGA-CS-HA (IC@PCH) nanoparticle (NP) synthesis procedure. (B) Schematic illustration of the effect of IC@PCH NPs: photothermal-chemotherapy mediates tumor cell pyroptosis via the caspase-1 signaling pathway. ICG, indocyanine green; CuET, copper(II) bis(diethyldithiocarbamate); PLGA, poly(lactic-*co*-glycolic acid); PVA, polyvinyl alcohol; CS, chitosan; HA; hyaluronic acid; MRI, magnetic resonance imaging; PAI, photoacoustic imaging; NIR, near-infrared; DAMPs, damage-associated molecular patterns; GSDMD-N, N-terminal fragment of GSDMD; PTT, photothermal therapy; FLI, fluorescence imaging.

Here, we report an ICG-CuET@PLGA-CS-HA (named IC@PCH) NP nanodrug delivery system, incorporating the chemotherapeutic drug CuET and the photosensitizer indocyanine green (ICG) (Fig. 1A). Studies suggest that chitosan (CS) is adhesive and positively charged, while HA specifically targets CD44-overexpressing 4T1 cells with high biocompatibility and nonimmunogenicity [27,28]. Thus, ICG-CuET@PLGA-CS NPs can attract HA to their surface via charge interaction, enhancing the particles’ tumor-targeting ability and extending their circulation time. After intravenous injection, the NPs bind to the CD44 receptor via HA and target aggregation at the tumor site. They disintegrate in the acidic microenvironment of the tumor, releasing CuET and ICG, and realize combined chemotherapy-photothermal therapy under laser irradiation for minimally noninvasive treatment of BC (Fig. 1B). Meanwhile, ICG and CuET endow NPs with photoacoustic (PA)–magnetic resonance (MR)–fluorescence imaging capabilities [29–31]. The NPs are targeted to reach the tumor cells, provide rich imaging information, and specifically suggest tumor lesions, promising to increase the cancer diagnosis rate and improve the prognosis. More importantly, we performed RNA sequencing (RNA-seq) and western blot to demonstrate that ICP@CH NP-mediated photothermal-chemotherapy treatment led to pyroptosis via the NLRP3/caspase-1 classical pathway. This demonstrates that this therapeutic modality is without dependent biological targets of TNBC and can effectively mediate cell death. In summary, this study reveals a superb strategy by creating an NP-based targeted delivery system for tumor microenvironment (TME)/laser-triggered photothermal-chemotherapy, effectively addressing the nonspecificity of endocrine/genetic treatment in TNBC.

## Materials and Methods

### Materials

ICG and polyvinyl alcohol (PVA) were obtained from Sigma-Aldrich (St. Louis, MO, USA). CuET was obtained from TCL (Shanghai, China). Carboxyl-PLGA (COOH–PLGA) (molecular weight 12,000 Da, 50:50) was obtained from Daigang Biomaterial (Jinan, China). CS and HA were purchased from Macklin (Shanghai, China). All other reagents and materials were directly obtained from the lab and were commercially available.

### Preparation of IC@PCH NPs

ICG-CuET@PLGA (IC@P) NPs and IC@PCH NPs were prepared using the double-emulsification method. In a 50-ml centrifuge tube, 50 mg of PLGA, 5 mg of CuET, and 2 ml of dichloromethane were added and processed using an ultrasonic oscillator for 30 s to dissolve them completely. Subsequently, 4 mg of ICG was dissolved in 200 μl of ultrapure water and added to the above mixture and emulsified using a sonicator (VCX130; Sonics and Materials, USA) at 100 W for 3 min to obtain the primary emulsion. An 8-ml volume of 5% (w:v) PVA was introduced into the primary emulsion and emulsified for 2 min to obtain a complex emulsion. To obtain the IC@P NP solution, isopropanol was added to the complex emulsion and evaporated with stirring to evaporate the dichloromethane. Finally, the NP solution was washed 3 times by high-speed centrifugation, resuspended in pure water, and stored in a 4 °C refrigerator protected from light.

To coat HA, we first prepared ICG-CuET@PLGA-CS NPs. The preparation procedure was the same as for IC@P NPs except that 8 ml of 5% PVA solution was replaced by 3 ml of 5% PVA solution and 3 ml of 2% CS, which had been dissolved in 3% acetic acid. Then, 10 mg of HA was added, shaken on a shaker for 1 h, washed 3 times, and resuspended in pure water to obtain an aqueous solution of IC@PCH NPs.

### Characterization of IC@PCH NPs

The morphological features of NPs were observed by scanning electron microscopy (Hitachi SU8010, Japan) and low-pressure transmission electron microscopy (TEM; FEI Tecnai G2 12, USA). The composition of the NPs was analyzed by an ultraviolet (UV)–visible–near-infrared (NIR) spectrometer (Shimadzu U4000, Japan), a Fourier transform infrared spectrometer (Nicolet iS10, USA), an x-ray diffractometer (Bruker D8 Advance, Germany), and an x-ray photoelectron spectrometer (Thermo Escalab 250XI, USA) to analyze the composition. The particle size and potential were analyzed by a particle size potentiostat, and the 1-week stability was analyzed.

### CuET release

Equal amounts of IC@PCH NP solutions were separately packed into dialysis bags (molecular weight cutoff 3,500, soluble) and randomly divided into 2 groups (*n* = 3). The samples of the 2 groups were placed in phosphate-buffered saline (PBS) buffer medium (30% ethanol, 0.02% sodium azide, and 0.01% Tween-80) at pH = 7.4 and pH = 6.5, respectively. A blue-necked bottle containing NPs and buffer medium was placed in a thermostatic oscillator, and 1 ml of buffer medium was collected at the time points of 0, 1, 2, 3, 4, 5, 6, 7, 8, 9, 10, 11, and 12 h. After each sample collection, another 1 ml of fresh buffer medium was added to the blue-necked bottle to ensure a constant volume of buffer medium. The samples obtained above were analyzed by high-performance liquid chromatography, and the cumulative release rate of CuET was deduced from the standard curve.

### In vitro photothermal effect of IC@PCH NPs

PBS and different concentrations of IC@PCH NPs (0.5, 1, 2, 4, and 6 mg/ml) were irradiated under an 808-nm laser (1.5 W/cm^2^) for 6 min to explore the effect of concentration on the warming effect (*n* = 3); 2 mg/ml IC@PCH NPs were irradiated under different intensities of an 808-nm laser (0.5, 1.0, 1.5, and 2 W/cm^2^) for 6 min to explore the effect of laser intensity on the warming effect (*n* = 3). A 10 μg/ml solution of ICG, CuET, and PLGA was exposed to a 1.5 W/cm^2^ laser to explore the source of the photothermal conversion performance of the NPs (*n* = 3). The IC@PCH NP solution and free ICG solution were exposed to a 1.5 W/cm^2^ laser 4 times to observe the effect of the laser irradiation and cooling process on the temperature. The warming process was recorded by infrared thermography.

### Cell lines

4T1 mouse BC cells and MCF-10A cells were purchased from Procell (Wuhan, China). Human umbilical vein endothelial cells (HUVECs) were obtained from the Department of Ultrasound and Chongqing Key Laboratory of Ultrasound Molecular Imaging. 4T1 cells and HUVECs were cultured in complete RPMI 1640 (Procell). MCF-10A cells were cultured in MCF-10A cell-specific medium (Procell). All cells were cultured in a 37 °C incubator with 5% CO_2_.

### Intracellular endocytosis of IC@PCH NPs

4T1 cells and MCF-10A cells were inoculated in laser copolymerization dishes for 24 h. Then, 2 mg/ml of IC@P-DiI NPs, and IC@PCH-DiI NPs were added to the 4T1 cell group and MCF-10A cell group, respectively, followed by coincubation for 1, 2, and 3 h. Each group was fixed by adding paraformaldehyde and then washed with PBS, and finally the cells (blue) were observed to phagocytose the NPs (red) under a laser confocal microscope (Nikon, Japan). Each group was incubated with different NPs, and then cells were washed, harvested, centrifuged, and resuspended. The cells were analyzed for phagocytosis in each group by flow cytometry analysis.

### In vitro safety evaluation and efficacy evaluation

HUVECs and MCF-10A and 4T1 cells were inoculated in 96-well plates and were incubated with different concentrations of IC@PCH NPs (0, 0.25, 0.5, 1, 2, and 5 mg/ml) for 24 h. The cellular activity was then determined by Cell Counting Kit-8 (CCK-8) assay.

4T1 cells were inoculated in 96-well plates, and they were divided into 9 groups, namely, the control (PBS) group, laser-only (PBS + laser) group, free CuET group, DSF@PLGA-CS-HA (D@PCH) NPs + CuCl_2_@PLGA-CS-HA (Cu^2+^@PCH) NPs group, IC@PCH NPs group, CuET@PLGA-CS-HA (C@PCH) NPs group, ICG@PLGA-CS-HA (I@PCH) NPs + laser group, IC@P NPs + laser group, and IC@PCH NPs + laser group. All drugs were used in 3 concentrations (0.5, 1, and 2 mg/ml), and the laser intensity was controlled at 1.5 W/cm^2^. After the treatment, the cell viability of each group was determined using the CCK-8 method.

4T1 cells were plated in a 12-well plate and incubated for 24 h before 9 treatments were applied. Following 3 washes with PBS, each well received 500 μl of calcein-AM and propidium iodide (PI) working solution to stain live (green) and dead (red) cells. After 30 min, 3 more washes were performed followed by observation under fluorescence microscopy (Olympus CKX53, Japan). 4T1 cells were plated in a 6-well plate and incubated for 24 h before 7 treatments. Posttreatment, cells were washed thrice with PBS, harvested via trypsin digestion, centrifuged at 2,000 rpm for 5 min, and resuspended in PBS containing annexin V–fluorescein isothiocyanate (5 μl) and PI (10 μl). Cells were then analyzed for apoptosis through flow cytometry (CytoFLEX, California, USA).

### Mechanistic investigation of IC@PCH NPs in inducing photothermal-chemotherapy

The 4T1 cells were treated with 2 mg/ml of IC@PCH NP solution as well as 1.5 W/cm^2^ of an 808-nm laser, and then the morphology of cells was observed under a light microscope. Next, 4T1 cells were divided into the control and IC@PCH NPs + laser groups (*n* = 3) and were subjected to transcriptome sequencing and bioinformatic analysis after treatment. Differentially expressed genes (DEGs) with fold change >2 and *P* value <0.05 were screened out. Functional enrichment analysis of DEGs was performed utilizing the Gene Ontology framework. In addition, the Kyoto Encyclopedia of Genes and Genomes (KEGG) was employed to pinpoint pathways that exhibited significant associations with the identified DEGs.

#### Western blot validation of the screened NLRP3 signaling pathway

4T1 cells were inoculated into 6-well plates and grouped for treatment. The groupings were control, laser-only, C@PCH NPs, I@PCH NPs + laser, and IC@PCH NPs + laser groups. Proteins were extracted from the cells and separated by sodium dodecyl sulfate–polyacrylamide gel electrophoresis. Then, they were transferred to a polyvinylidene fluoride membrane (Bio-Rad, USA) and blocked. Target proteins were detected using specific antibodies, including NLRP3, caspase-1, cleaved caspase-1, GSDMD, N-terminal fragments of GSDMD (GSDMD-N), and β-actin (Proteintech). Stained bands were observed by a chemiluminescent detection system and analyzed in terms of grayscale intensity utilizing the ImageJ software. The scheme illustrating the mechanism was drawn using BioRender.

### Animals

All animal experiment protocols were approved by Institutional Animal Care and Use of Chongqing Medical University (approval number: IACUC-CQMU-2024-0594). Female BALB/c mice (8 weeks) were obtained from the Laboratory Animal Center of Chongqing Medical University; 4 × 10^6^ 4T1 cells were dispersed in a 100-μl solution and injected into the right dorsal side of BALB/c mice to establish a mouse hormonal tumor model. Treatment was started when the tumor grew to 45 to 75 mm^3^. The tumor volume was calculated using the formula [1/2 × length × (width)^2^].

### In vivo biosafety

Eight-week-old female BALB/c mice (*n* = 15) were selected and grouped according to 20 d–15 d–10 d–5 d–pre time points (*n* = 3 per time). Each group was injected with 8 mg/ml NPs through the tail vein at a dose of 200 μl each. Animal blood collection operation was carried out after 20 d for routine blood (white blood cells, lymphocytes, red blood cells, hemoglobin, mean corpuscular volume, and platelets) and blood biochemistry examination (alanine aminotransferase, aspartate aminotransferase, urea, creatine kinase, creatine kinase-MB, and lactate dehydrogenase 1). The major organs of mice were collected for hematoxylin–eosin (H&E) staining.

### In vivo photothermal capacity and photothermal-chemotherapy

The BALB/c mice were divided into the laser-only (PBS + laser) group, IC@P NPs + laser group, I@PCH NPs + laser group, and IC@PCH (ICG-CuET @PLGA-CS-HA) NPs + laser group. After the mice in each group were injected with NPs (5 mg/ml, 200 μl) for 2 h, the tumors were irradiated with a 1.5 W/cm^2^ laser for 6 min, and the warming process of the tumor site was recorded by using an infrared thermography camera.

Twenty-eight mice were randomly divided into 7 groups: the control (PBS) group, laser-only (PBS + laser) group, D@PCH NPs + Cu^2+^@PCH NPs group, IC@PCH NPs group, C@PCH NPs group, I@PCH NPs + laser group, and IC@PCH NPs + laser group, with 4 mice per group. Two hours after tail vein injection (5 mg/ml, 200 μl), the tumors were irradiated with an 808-nm laser (1.5 W/cm^2^) for 6 min, and the entire course of treatment was performed only once. The tumor size and body weight of the mice were measured every other day throughout the treatment cycle. The mice were executed after 20 d of treatment, and the tumors were collected for H&E staining, terminal deoxynucleotidyl transferase dUTP nick end labeling (TUNEL) fluorescence staining, and proliferating cell nuclear antigen (PCNA) fluorescence staining.

### In vitro and in vivo multimodality imaging

CuET, ICG, and IC@PCH NP solutions (1.2 mg/ml) were injected into the agarose gel model, respectively, at 200 μl per well. The optimal excitation wavelength was explored by irradiating with a 690- to 960-nm laser using a PA imager (Visual Sonics Inc., Toronto, Canada). Different concentrations (0.4, 0.6, 0.8, 1.0, and 1.2 mg/ml) of IC@PCH NP solutions were injected into the agarose gel model and were irradiated by the laser at the optimal excitation wavelength to observe the PA signal values. Different concentrations (0.4, 0.6, 0.8, 1.0, and 1.2 mg/ml) of Cu^2+^ and IC@PCH NP solutions were injected into the agarose gel model, and the MR signal values were recorded. Different concentrations (0.4, 0.6, 0.8, 1.0, and 1.2 mg/ml) of IC@PCH NP solutions were injected into 48-well plates, and the fluorescence signal values were observed. IC@P NP, IC@PCH NP, ICG@PCH NP, and CuET@PCH NP solutions (5 mg/ml, 200 μl) were injected into mice through the tail vein, and PA imaging of the tumor site was performed before and 1 h–2 h–3 h–6 h–12 h after the injection, and the signal values of the tumor area were recorded. IC@P NP and IC@PCH NP solutions (5 mg/ml, 200 μl) were injected into mice through the tail vein, and magnetic resonance imaging (MRI) of mice was performed before and 1 h–2 h–3 h–6 h–12 h after the injection, and the signal values of the tumor area were recorded. IC@P NP and IC@PCH NP solutions (5 mg/ml, 200 μl) were injected into mice, and PA imaging of the tumor site was performed before and 0.5 h–1 h–2 h–3 h–6 h after the injection, and the signal values of the tumor area were recorded.

### Biodistribution in vivo

IC@P NPs and IC@PCH NPs (5 mg/ml, 200 μl) were injected into 4 T1 hormonal mice through the tail vein. Major organs (including the heart, liver, spleen, lungs, and kidneys) and tumors were collected 24 h after injection. The biodistribution of IC@P NPs and IC@PCH NPs was measured ex vivo using a Xenogen IVIS spectral imaging system (IVIS Lumina III, PerkinElmer, Waltham, MA).

### Statistical analysis

All data were analyzed using GraphPad Prism 9.0, presented as mean ± standard deviation (SD). Calculations were performed using a 2-tailed Student *t* test. *P* values less than 0.05 represent statistically significant differences.

## Results and Discussion

### Preparation and characterization of IC@PCH NPs

**Fig. 2.**
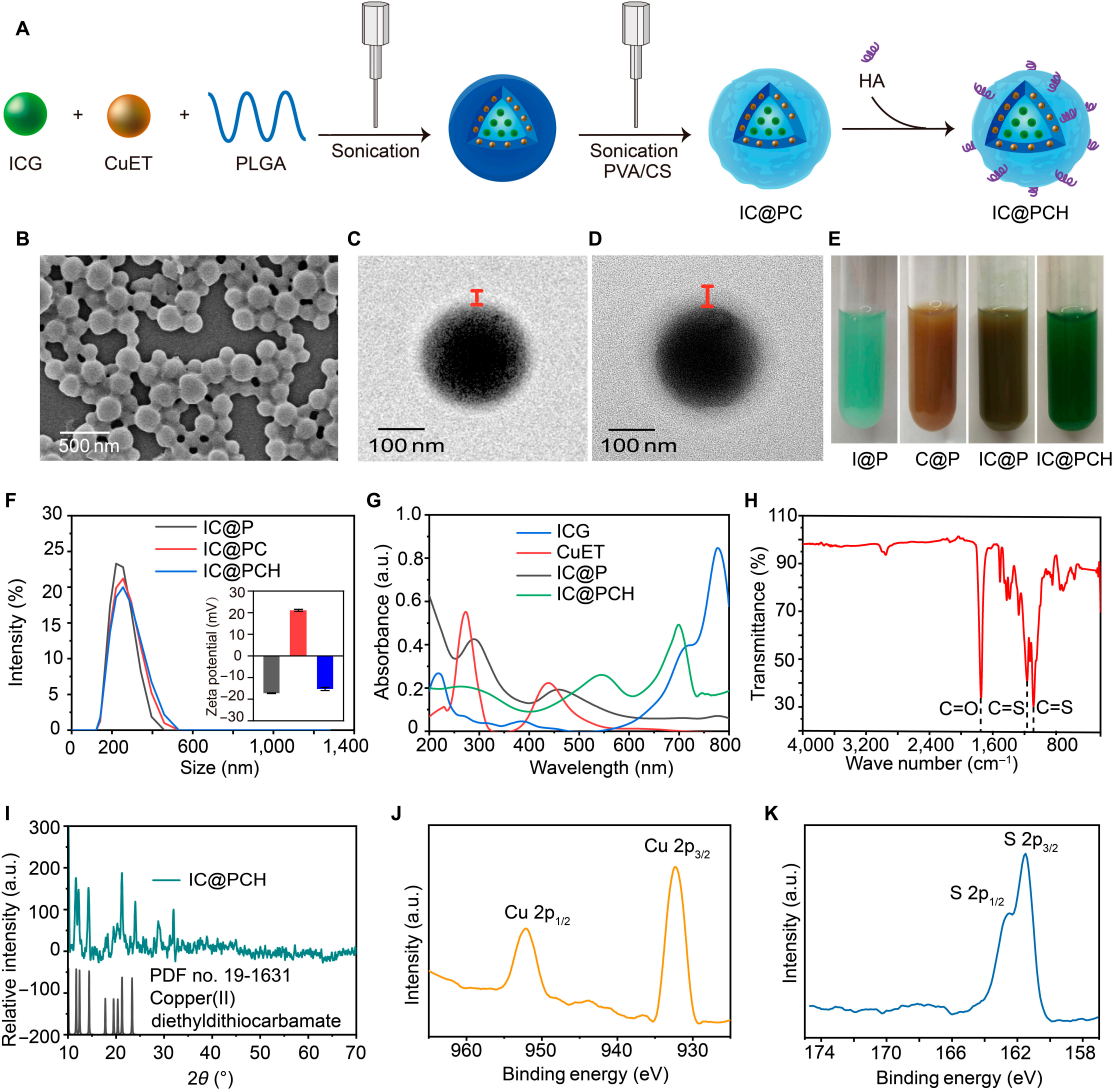
Synthesis and characteristics of IC@PCH NPs. (A) A schematic illustration depicting the synthetic pathway of IC@PCH NPs. Scanning electron microscopy (SEM) images of (B) IC@PCH NPs. Transmission electron microscopy (TEM) images of (C) ICG-CuET@PLGA (IC@P) NPs and (D) IC@PCH NPs. (E) Digital images of I@P NP, C@P NP, IC@P NP, and ICP@CH NP solutions. (F) Size distribution and *ζ* potential (inset) of IC@P NPs, IC@PC NPs, and IC@PCH NPs. (G) Ultraviolet–visible–near-infrared (UV–Vis–NIR) absorption spectra of ICG solution, CuET solution, IC@P NPs, and IC@PCH NPs. (H) Fourier transform infrared (FTIR) spectra of IC@PCH NPs. (I) X-ray diffraction (XRD) of IC@PCH NPs and CuET. (J and K) High-resolution Cu 2p and S 2p XPS spectra of IC@PCH NPs.

IC@PCH NPs were successfully synthesized using a simple 2-emulsion method, and the synthesis process is shown in Fig. 2A. These IC@PCH NPs exhibit spherical morphology with a smooth surface and a uniform particle size as depicted by scanning electron microscopy (Fig. 2B). A more obvious halo ring surrounding the surface of IC@PCH NPs is observed under TEM compared to IC@P NPs (Fig. 2C and D), verifying successful HA/CS decoration. Figure 2E reveals that the I@P NPs appear green, C@P NPs are brownish, and IC@PCH NPs and IC@P NPs exist somewhere between these 2 color schemes, signifying the successful incorporation of both IC@PCH and IC@P with both ICG and CuET. IC@PCH NPs exhibit superior greenness compared to IC@P NPs, suggesting an increase in ICG loading after HA/CS incorporation, possibly due to the negative charge of ICG being attracted by the positive CS. The particle size potentiostat results showed that the particle size of the IC@P NPs was about 219.8 nm (polydispersity index [PDI] = 0.048), and the size was increased to 248.4 nm (PDI = 0.108) and 259.2 nm (PDI = 0.119) sequentially after loading of CS and HA, which was in agreement with the TEM results (Fig. 2F and Table S1). In pure aqueous solution at pH 7.4, the *ζ* charge of IC@P NPs was −17.67 mV, which changed to 21.57 mV after loading CS and reversed to −16.17 mV after continued loading of HA, indicating that HA was loaded by positive and negative charge attraction (Fig. 2F).

Subsequently, we analyzed the drug loading performance of IC@PCH NPs using UV spectroscopy. The results showed that the IC@PCH NPs had obvious absorption peaks at 263, 545, and 699 nm, which proved the successful loading of ICG and CuET (Fig. 2G). The UV absorption peaks of ICG were shifted from 713 and 780 nm to 545 and 699 nm, and the UV absorption peaks of CuET were shifted from 437 to 263 nm, which may be due to the spatial site resistance effect [32]. Based on the UV spectroscopy and standard curves of ICG and CuET (Fig. S1A to D), it was determined that the encapsulation rate of ICG in IC@PCH NPs was about 52.40% with a loading rate of about 7.33%, and the encapsulation rate of CuET in IC@PCH NPs was about 34.40% with a loading rate of about 6.01%. In addition, the successful incorporation of CuET was confirmed through Fourier transform infrared spectroscopy, which exhibited characteristic peaks in the range of 1,020 to 1,200 cm^−1^ (C=S), indicative of the stretching vibrations associated with CuET (Fig. 2H). This analysis provides additional verification of the successful loading process. The absorption peak of the unsaturated group C=O double bond at 1,747 cm^−1^ was from PLGA. Notably, no markedly changes were detected between the x-ray diffraction patterns of IC@PCH and CuET, suggesting that the crystal structure of CuET remained unchanged after being loaded into NPs (Fig. 2I). Through x-ray photoelectron spectroscopy (XPS), we assessed the chemical state of Cu and S. The binding energy centers at 932.3 and 952.8 eV correspond to high-resolution XPS peaks of Cu 2p_3/2_ and Cu 2p_1/2_, respectively (Fig. 2J). The binding energy centers of S 2p_3/2_ and S 2p_1/2_ were located at 161.5 and 162.5 eV, respectively, which indicated the presence of sulfides and disulfides (Fig. 2K). These findings align with the XPS data of CuET nanocrystals.

The stability and responsiveness of IC@PCH NPs to the release of CuET were particularly to be verified in subsequent experiments. After storing the aqueous solution of IC@PCH NPs at 4 °C for 1 week, no significant change in average particle size or potential was observed, and no aggregation or precipitation occurred within 7 d (Fig. S2A), highlighting the exceptional long-term stability of the NPs obtained. NPs’ release of CuET is significantly faster in pH 6.5 buffer compared to its release in a typical neutral pH environment (pH = 7.4) (Fig. S2B). This is owing to PLGA’s solubility under acidic conditions [33]. Therefore, NPs can release drugs triggered by the TME and thus exhibit antitumor activity. Meanwhile, this slow-release property facilitates the continuation of chemotherapy at the tumor site.

### In vitro photothermal effect of IC@PCH NPs

**Fig. 3.**
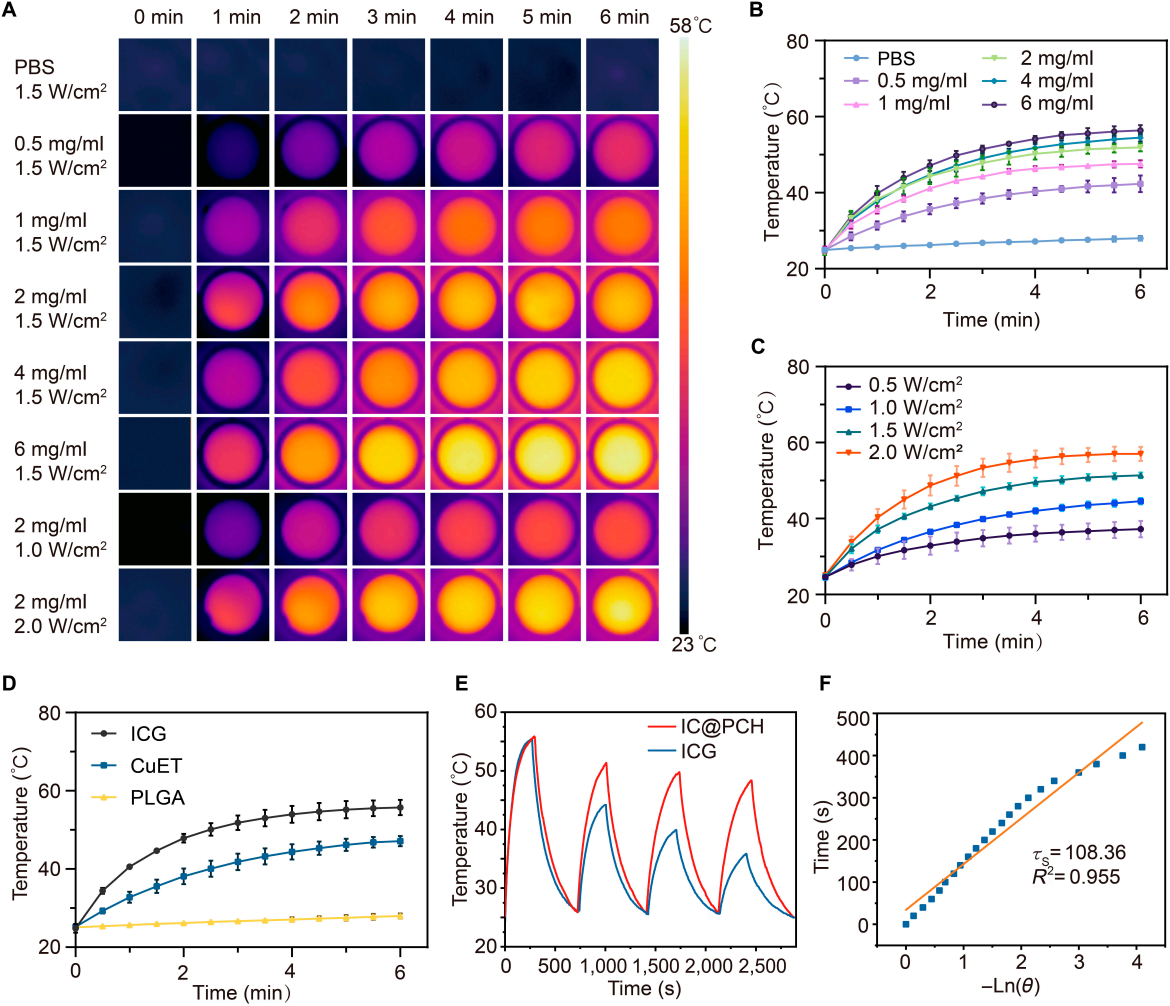
In vitro photothermal performance of IC@PCH NPs. (A) Infrared thermograms of IC@PCH NPs under 808-nm laser irradiation at different concentrations, powers, and times. (B) Heating curves of varying concentrations of IC@PCH NPs by 1.5 W/cm^2^. (C) Heating curves of IC@PCH NP aqueous dispersion (2 mg/ml) exposure at varying power densities. (D) ICG, CuET, and PLGA’s photoheating curves at 10 μg/ml concentration. (E) Heating curve of IC@PCH NPs and ICG aqueous dispersion under 4 cycles of heating and subsequent cooling processes. (F) The time constant was derived from the duration of the cooling phase within these cycles. PBS, phosphate-buffered saline.

Given ICG’s high absorption in the NIR region, this study examined the photothermal conversion performance of IC@PCH NPs in an aqueous solution. The infrared thermograms at varying IC@PCH NP concentrations and laser powers are shown in Fig. 3A. Then, Fig. 3B shows that the temperature of the IC@PCH NP aqueous solution significantly increases upon 808-nm laser irradiation (1.5 W/cm^2^, 6 min), exhibiting a concentration-dependent pattern. Furthermore, evident laser power dependence appeared during NIR irradiation for 6 min, suggesting easy control of sample temperature by adjusting laser power (Fig. 3C). The heat curve analysis suggested that IC@PCH NP’s photothermal properties are primarily driven by ICG, with limited heating effect from CuET (Fig. 3D). Furthermore, compared to free ICG, IC@PCH NPs exhibited superior photothermal stability during 4 heating and cooling cycles, possibly due to the enhanced photostability provided by coating modifications of ICG and CuET in polymer materials (Fig. 3E). Through calculations, the photothermal conversion efficiency of IC@PCH NPs was calculated at 20.23% (Fig. 3F). These findings suggest that as a photothermal agent, IC@PCH NPs efficiently convert light energy into thermal energy.

### Intracellular endocytosis of IC@PCH NPs

Utilizing confocal laser scanning microscopy, we evaluated the cellular uptake efficiency of IC@PCH and ICP NPs, which were decorated with 1,1′-dioctadecyl-3,3,3′,3′-tetramethylindocarbocyanine perchlorate (DiI, red), and cell nuclei were identified using 4′,6-diamidino-2-phenylindole (blue). Despite ICG’s inherent fluorescence, DiI was utilized because it is within the range of confocal laser scanning microscopy and flow cytometry excitation/emission wavelengths (the ICG emission wavelength is 780 nm, the excitation wavelength is 820 nm, the DiI emission wavelength is 565 nm, and the excitation wavelength is 549 nm) [34]. The CD44 receptor is a cell surface glycoprotein receptor with dual roles and expression during cellular processes and is particularly important for the targeting properties of HA-coated NPs [35]. Under normal physiological conditions, CD44s are predominantly expressed and involved in the maintenance of tissue homeostasis and repair functions (e.g., low levels of CD44 expression in HUVECs play a protective role by stabilizing the endothelial barrier), whereas under pathological conditions (e.g., TNBC), expression of CD44v6 is significantly up-regulated, promoting disease progression by driving epithelial–mesenchymal transition and tumor stem cell properties [36,37]. Molecularly, the Link module of the CD44 receptor binds specifically to the hexasaccharide unit of HA via a hydrogen bonding network [28]. These molecular characteristics directly informed our NP design. Experiments included 4 groups: (1) MCF-10A cells + IC@P NPs, (2) MCF-10A cells + IC@PCH NPs, (3) 4T1 cells + IC@P NPs, and (4) 4T1 cells + IC@PCH NPs.

**Fig. 4.**
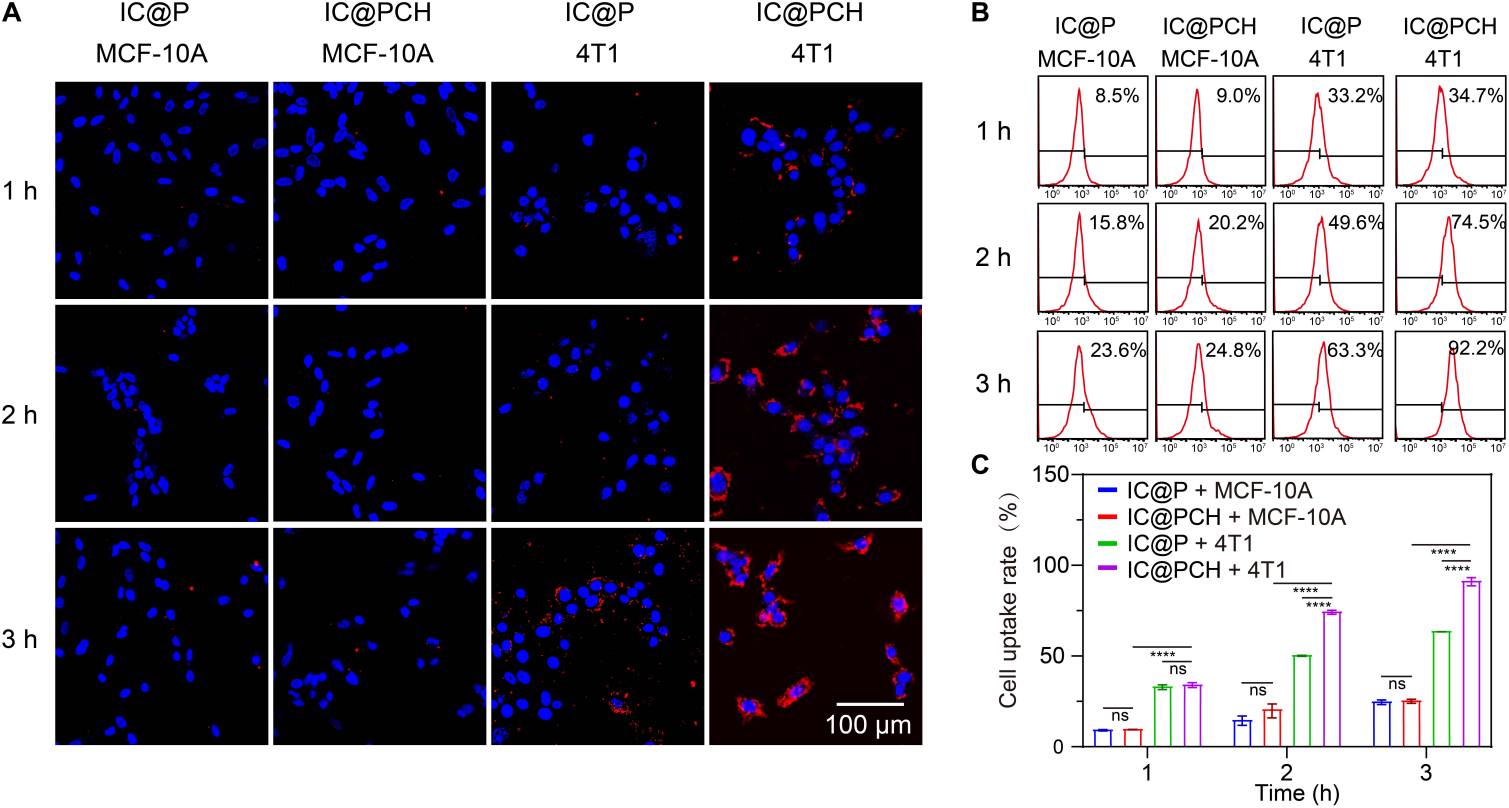
Intracellular endocytosis of IC@PCH NPs. (A) CLSM analysis (scale bars: 100 μm) and (B) FCM analysis of intracellular uptake of DiI-labeled IC@P and IC@PCH NPs in 4T1 and MCF-10A cells for 1, 2, and 3 h. (C) Quantitative analysis of (B) (*n* = 3). The results are presented as mean ± standard deviation (SD). ****P < 0.0001. ns, not significant; CLSM, confocal laser scanning microscopy; FCM, flow cytometry; DiI, 1,1′-dioctadecyl-3,3,3′,3′-tetramethylindocarbocyanine perchlorate.

As incubation time advanced, the red dots representing NPs were progressively engulfed by cells. After 3 h of culture, DiI-labeled IC@PCH NPs rapidly congregated around the membrane of the group 4 4T1 cells and infiltrated within these. Only limited IC@P NPs were observed to accumulate on MCF-10A cells in group 3. Conversely, no significant aggregation of IC@PCH NPs was detected near MCF-10A cells in group 1 and group 2 (Fig. 4A). This may be attributed to the ligand–receptor interaction between HA-coated NPs and CD44-receptor-overexpressing 4T1 cells. Meanwhile, there was no specific binding of receptor ligands in groups 1, 2, and 3, but NPs were endocytosed more in the IC@P NPs + 4T1 cell group than in the MCF-10A cells, probably due to the more vigorous metabolism and active phagocytosis process in tumor cells. Flow cytometry data (Fig. 4B and C) show that after 3 h, the fluorescence intensity of IC@PCH NPs in 4T1 cells is 90.99%, compared to 57.08% for IC@P NPs. The fluorescence intensity of IC@P NPs and IC@PCH NPs in MCF-10A cells at the third hour was only 25.85% and 30.11%, respectively. These findings suggest the CD44 receptor’s critical role in the endocytosis of HA-functionalized NPs in 4T1 cancer cells. IC@PCH NPs can target cancer cells overexpressing CD44 receptors, potentially facilitating targeted cancer therapy.

### In vitro photothermal-chemotherapy treatment against 4T1 cells

**Fig. 5.**
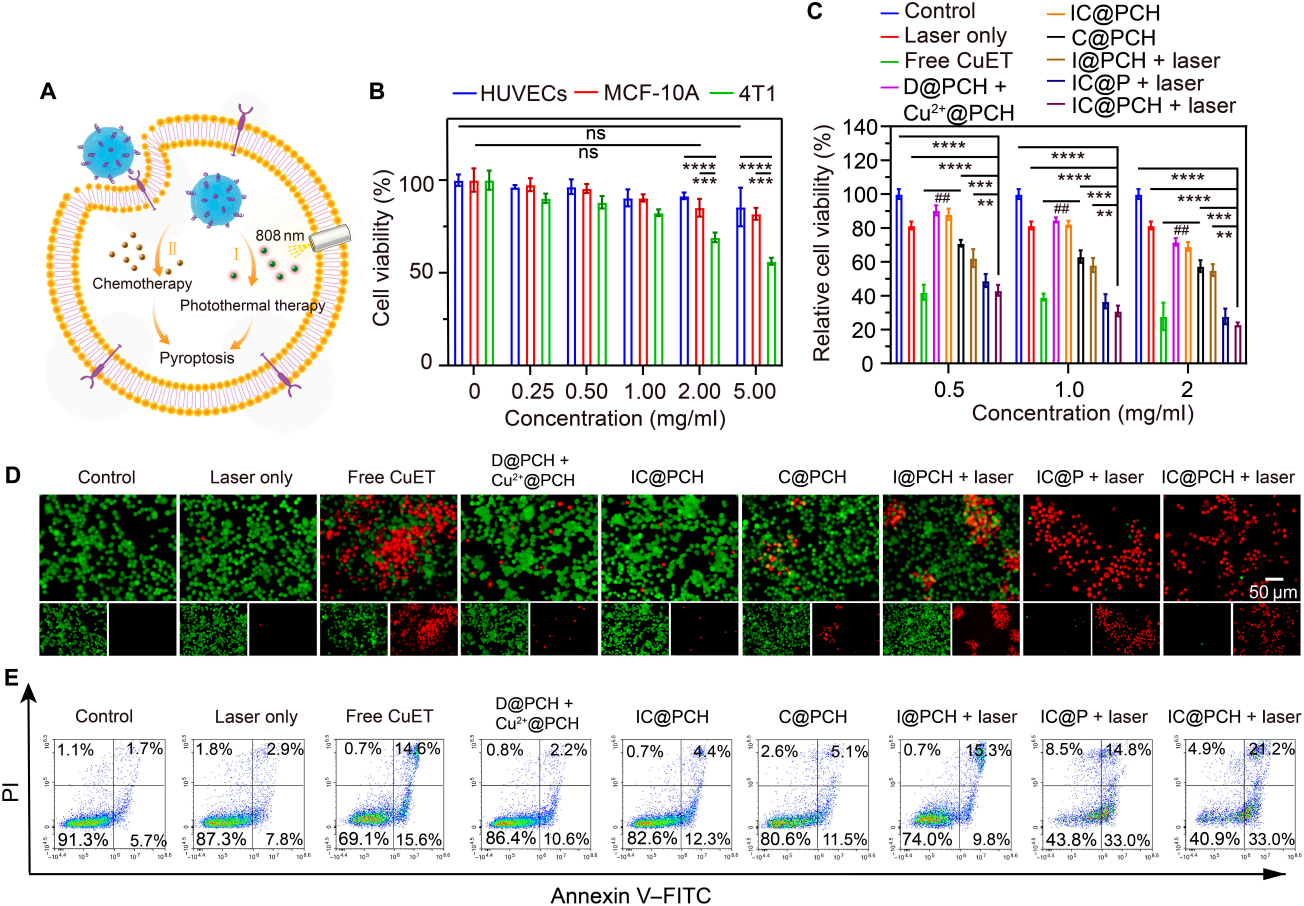
In vitro photothermal chemotherapy treatment against 4T1 cells. (A) A schematic illustration depicting the utilization of IC@PCH NPs for photothermal chemotherapy treatment against 4T1 cells. (B) Cytotoxicity against human umbilical vein endothelial cells (HUVECs) and MCF-10A and 4T1 cells after incubation with IC@PCH NPs for 24 h; *n* = 3. (C) The anticancer efficacy of various therapies against 4T1 cells; *n* = 3. (D) Live and dead fluorescence images of 4T1 cells in different treatment groups (scale bar: 50 μm). (E) Flow cytometry apoptosis assay stained with annexin V–fluorescein isothiocyanate (FITC) and propidium iodide (PI) after varying treatments. The results are presented as mean ± SD. **P < 0.01; ***P < 0.001; ****P < 0.0001.

Cell viability was examined by standard CCK-8 assay to validate IC@PCH NPs for the safety and efficacy of photothermal-chemotherapy treatment in vitro (Fig. 5A). As shown in Fig. 5B, there was no significant decrease in the viability of HUVECs after 24 h of coincubation with 5 mg/ml IC@PCH NPs compared with that of the control group, indicating that the injection of 5 mg/ml IC@PCH NPs was almost nontoxic to the blood vessels and had good biocompatibility. Compared with the control group, MCF-10A cells showed no significant decrease in survival after 24 h of coincubation with 2 mg/ml IC@PCH NPs, indicating that 2 mg/ml ICP@CH NPs at the tumor treatment site had no significant toxicity to normal breast cells. Exposure to 4T1 cells at IC@PCH NP concentrations of 2 and 5 mg/ml reduced their viability to 69.14% and 56.24% respectively, revealing potential tumor cell toxicity dependent on NP concentration. Meanwhile, as 4T1 cells show a higher level of CD44 expression compared to normal cells, the endocytosis of IC@PCH NPs by 4T1 cells is promoted, thereby resulting in a greater killing potency. Next, the anticancer effect of each treatment group on 4T1 cells was evaluated. As illustrated in Fig. 5C, the cell viability in the IC@PCH NPs + laser group was lower than that in both the C@PCH NPs (no ICG) and I@PCH NPs (no CuET) + laser groups. This indicates a synergistic anticancer effect of photothermal therapy and chemotherapy. Under laser irradiation, the cytotoxicity of IC@PCH NPs is higher than that of IC@P NPs, indicating that targeting NPs enhances anticancer efficacy. This could be attributed to the CD44-receptor-mediated internalization of more IC@PCH NPs by 4T1 cells, thereby potentiating photothermal therapy and chemotherapy efficacy. Given the superior biosecurity of the 808-nm laser, only the laser group exhibited low cytotoxicity. Due to the direct use of CuET skipping the reaction loss between DSF and Cu^2+^, the cell survival rate of the C@PCH group is lower than that of the D@PCH NPs + Cu^2+^@PCH NPs group. The higher cell survival of the IC@PCH NPs group compared to that of the IC@PCH NPs + laser group demonstrates the good safety and controllability of IC@PCH NPs; i.e., the strong antitumor properties are triggered after laser irradiation. Compared to IC@PCH NPs, C@PCH NPs have higher cytotoxicity because their CuET loading rate is higher, resulting in more CuET entering tumor cells. Additionally, short-term tumor cell toxicity was reduced with C@PCH NPs compared to that with the free CuET group, likely due to PLGA nanoshell’s pH-dependent sustained release property, conferring a milder yet enduring antitumor capacity. Lastly, similar results were obtained using different concentrations of NPs (0.5, 1, and 2 mg/ml), with the efficacy escalating with concentration.

As shown in the fluorescence microscope image (Fig. 5D), both the C@PCH NPs and I@PCH NPs + laser groups revealed modest red fluorescence, suggesting limited cytotoxicity via chemo + targeted treatment, photothermal + targeted treatment. The prominent red fluorescence signal was observed when 4T1 cells were co-cultured with IC@P NPs or IC@PCH NPs followed by laser irradiation. Comparable outcomes were noted in the annexin V–fluorescein isothiocyanate/PI staining assay (Fig. 5E). Laser-irradiated IC@P NPs and IC@PCH NPs evoke high levels of apoptosis, with the cytotoxic potency of IC@PCH NPs marginally higher than that of IC@P NPs. These findings validate the necessity of a photothermal + chemo + targeted strategy for tumor eradication. It should be noted that although IC@P NPs and IC@PCH NPs showed similar killing ability in tumor cells, considering the biocompatibility and targeting of HA, IC@PCH NPs may be more advantageous in in vivo circulation.

### Mechanistic investigation of IC@PCH NPs in inducing photothermal-chemotherapy

**Fig. 6.**
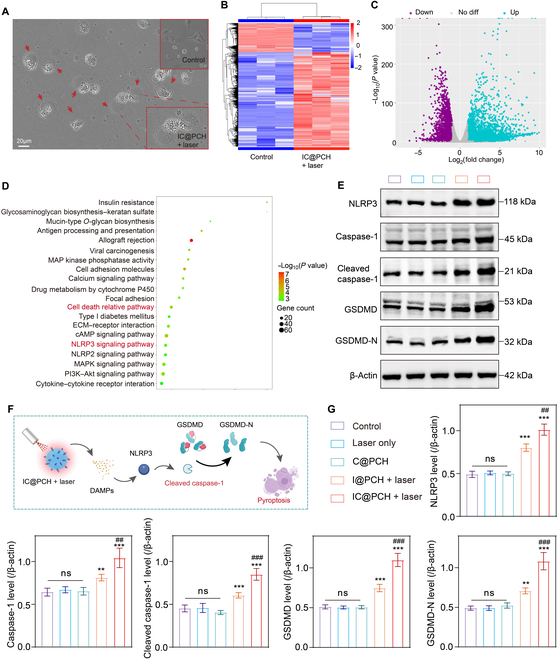
Mechanistic investigation of IC@PCH NPs in inducing photothermal chemotherapy. (A) Light micrograph of 4T1 cells treated with PBS and IC@PCH NPs + laser (scale bar: 20 μm). (B) Heat map of the differentially expressed genes between the control and IC@PCH NPs + laser groups. Red and blue indicate the up-regulated and down-regulated genes. (C) Volcano plot of genes that were differentially expressed between the control and IC@PCH NPs + laser groups. (D) Representative Kyoto Encyclopedia of Genes and Genomes (KEGG) pathways associated with genes that were significantly differentially expressed between the control and IC@PCH NPs + laser groups. (E) The expression levels of NLRP3, caspase-1, cleaved caspase-1, GSDMD, and N-terminal fragments of GSDMD (GSDMD-N) in 4T1 tumors after various treatments detected by western blot. (F) A scheme illustrating the mechanism of photothermal-chemotherapy-induced pyroptosis. (G) Quantitative analysis of (E); *n* = 3. The results are presented as mean ± SD. **P < 0.01; ***P < 0.001; ^##^*P* < 0.01; ^###^*P* < 0.001. MAP, mitogen-activated protein; ECM, extracellular matrix; cAMP, cyclic adenosine 3,5-monophosphate; PI3K, phosphoinositide 3-kinase.

It is worth noting that the cells after treatment showed a variety of morphological damage of tumor cells mediated by IC@PCH NPs, and the “balloon-like” changes were observed under the light microscopy, and the cells were enlarged and burst (Fig. 6A). To further investigate the mechanism of action of IC@PCH NP-mediated photothermal-chemotherapy, RNA-seq and bioinformatic analysis of 4T1 cells in the control group and IC@PCH NPs + laser group was performed. In the heat map, blue denoted genes that were down-regulated, while red indicated genes that were up-regulated (Fig. 6B). The volcano plot displayed that, in comparison to the control group, the IC@PCH NPs + laser group exhibited significant up-regulation of 547 genes and notable down-regulation of 668 genes (log_2_FC ≥ 1, *P* ≤ 0.05 for up-regulation; log_2_FC ≤ −1, *P* ≤ 0.05 for down-regulation; otherwise no differential expression) (Fig. 6C). To better understand the major metabolic and signaling pathways of DEGs in the IC@PCH NPs +laser group, we conducted KEGG pathway enrichment analysis. The results highlighted the involvement of insulin resistance, allograft rejection, cell-death-related pathways, and the NLRP3 signaling pathway in the therapeutic mechanism of IC@PCH NPs (Fig. 6D). Some studies have shown that the NLRP3 signaling pathway is closely related to a new mode of death, which is pyroptosis [38,39].

Pyroptosis is programmed cell death mediated by the pore-forming protein gasdermin and is closely associated with the inflammatory response [40]. Pattern-recognition receptors such as toll-like receptors and nucleotide oligomerization domain-like receptors recognize intra- and extracellular signals and form inflammatory vesicles assembled with pro-caspase-1 and apoptosis-associated speck-like protein to activate caspase-1. Activated caspase-1 cleaved GSDMD into GSDMD-N. GSDMD-N nonselectively perforates membrane pores in the cell membrane, and the inflammatory factors were secreted, ultimately causing cell swelling, lysis, and death [41,42]. The light microscopy and RNA-seq results suggested that IC@PCH NPs may mediate 4T1 cell death through the pyroptosis pathway (Fig. 6F). To validate the above results and understand the mechanism of IC@PCH NP-mediated pyroptosis, the NLRP3, caspase-1, cleaved caspase-1, GSDMD, and GSDMD-N protein expression levels in 4T1 cells with different treatments were evaluated by western blot analysis (groups: control group, laser-only group, C@PCH NPs group, I@PCH NPs + laser group, and IC@PCH NP + laser group) (Fig. 6E). As expected, the levels of key proteins for pyroptosis were significantly elevated in the I@PCH NPs + laser group and IC@PCH NP + laser group (Fig. 6G), which was attributed to the predominance of photothermal effects in the treatment. Meanwhile, the expression level of pyroptosis key proteins was significantly higher in the IC@PCH NP + laser group than in the I@PCH NPs + laser group, proving the chemotherapeutic agents’ effective synergism. Therefore, we concluded that IC@PCH NPs could inhibit tumor cell proliferation by initiating a classical caspase-1-dependent pathway leading to tumor cell pyroptosis. During treatment, TNBC often has no response to conventional therapy, while pyroptosis can bypass the “treatment blockade” and find new therapeutic targets. This makes IC@PCH NP-induced photothermal-chemotherapy a promising cancer treatment strategy.

### In vivo biosafety

**Fig. 7.**
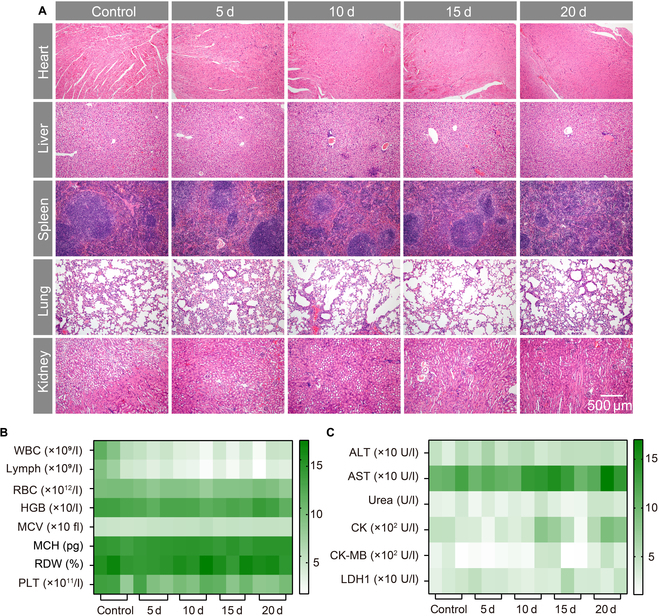
Biosafety of IC@PCH NPs in vivo. (A) Hematoxylin–eosin (H&E) staining of the main organs (heart, liver, spleen, lung, and kidney) in mice sacrificed at different time intervals (0, 5, 10, 15, and 20 d) after intravenous injection of IC@PCH NPs; the scale bars are 500 μm. (B) Blood routine and (C) blood biochemistry of mice at different time points after intravenous injection of IC@PCH NPs; *n* = 3. WBC, white blood cells; Lymph, lymphocytes; RBC, red blood cells; HGB, hemoglobin; MCV, mean corpuscular volume; PLT, platelets; MCH, mean corpuscular hemoglobin; RDW, red cell distribution width; ALT, alanine aminotransferase; AST, aspartate aminotransferase; CK, creatine kinase; CK-MB, creatine kinase-MB; LDH1, lactate dehydrogenase 1.

Using a novel biomedical nanomaterial in vivo requires first verification of its biosafety. An evaluation of the biological safety of IC@PCH NPs was conducted comprehensively. Healthy BALB/c mice were administered IC@PCH NPs (8 mg/ml, 200 μl) via intravenous injection on days 5, 10, 15, and 20, with a control group receiving saline. Blood and major organs (including the heart, liver, spleen, lungs, and kidneys) from these mice were collected for analysis. H&E staining of major organs showed little histopathologic change (Fig. 7A). Furthermore, biochemical and hematological analyses indicated no significant differences between groups at each time point (Fig. 7B and C), indicating the high biosecurity of IC@PCH in subsequent treatments.

### In vivo photothermal properties and in vivo treatment of hormonal mice

The synergistic in vivo targeted anticancer efficacy of photothermal and chemotherapeutic treatments with IC@PCH NPs was evaluated.

**Fig. 8.**
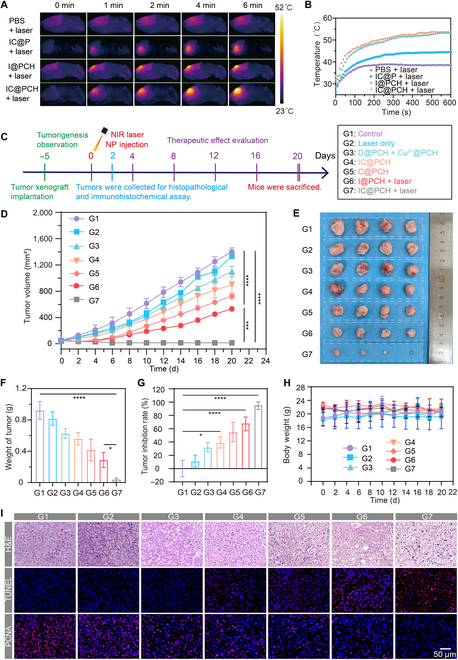
In vivo therapeutic efficacy of IC@PCH NPs. (A) Infrared thermographic images and (B) heating curves following tail vein injection of PBS, IC@P NPs, I@PCH NPs, and IC@PCH NPs under 808-nm laser irradiation (1.5 W/cm^2^). (C) Schematic of intratumor treatments. (D) Tumor volume growth trajectories for the control, laser-only, C@PCH NPs, I@PCH NPs + laser, and IC@PCH NP + laser groups posttreatment. (E) Pictures of the dissected tumors from each treatment group. (F) Tumor weight, (G) tumor inhibition rate, and (H) body weights of hormonal mice. (I) H&E staining, proliferating cell nuclear antigen (PCNA), and terminal deoxynucleotidyl transferase dUTP nick end labeling (TUNEL) immunohistochemistry staining in the tumor region in various treatment groups (scale bars: 50 μm). The data are shown as mean ± SD, *n* = 4 per group. *P < 0.05; ***P < 0.001; ****P < 0.0001.

As shown in Fig. 8A and B, the laser-only group experienced a slight increase in temperature to 38.5 °C, below the heat therapy threshold of 42 °C. This finding suggests that the NIR laser is safe for normal tissues and suitable for future clinical applications. The temperature of IC@PCH NPs and I@PCH NPs rapidly increased to 53.5 and 53.4 °C, respectively, upon laser irradiation, indicating that the efficacy of NP photothermal therapy is mainly derived from ICG. Moreover, compared to IC@PCH NPs, the temperature of the IC@P NPs + laser group was only 44.5 °C. This indicates that more ICG accumulated in the tumor region due to the superior targeting ability of the NPs coated with HA and higher ICG encapsulation rate, resulting in higher temperatures.

The protocol of intratumor treatments is sketched out in Fig. 8C. To further assess the synergistic anticancer effect, when the tumor volume grew to 45 to 75 mm^3^, 4T1 hormonal mice were randomly divided into 7 groups (*n* = 4): the control group, laser-only group, D@PCH NPs + Cu^2+^@PCH NPs group, IC@PCH NPs group, C@PCH NPs, I@PCH NPs + laser, and IC@PCH NP + laser groups. After the mice in each group were injected with the corresponding drug intravenously (5 mg/ml, 200 μl) for 2 h, the tumor area of the corresponding group was irradiated with NIR laser at 1.5 W/cm^2^ (808 nm) for 6 min (Fig. 8A). Tumor volumes were measured every 2 d (Fig. 8D). The IC@PCH NPs + laser group significantly inhibited tumor growth, the I@PCH NPs + laser group and the C@PCH NPs group exhibited moderate inhibition, while the D@PCH NPs + Cu^2+^@PCH NPs group and the IC@PCH NPs group slowed down the growth rate of the tumor only mildly. In contrast, the control and laser-only groups displayed notable tumor volume increases, suggesting no therapeutic effects. Tumor growth inhibition was more pronounced in the C@PCH NPs group than in the D@PCH NPs + Cu^2+^@PCH NPs group, suggesting that direct delivery of CuET helps to overcome the limitation that the DSF–Cu^2+^ delivery nano-delivery system is unable to efficiently form the antitumor active ingredients. The IC@PCH NPs + laser group had better treatment results compared to the IC@PCH NPs group, again demonstrating the good laser control properties of IC@PCH NPs. Harvested tumor weights and in vivo tumor suppression rates from each treatment group further confirmed these results (Fig. 8E to G). Additionally, there were no significant differences in body weight among the groups (Fig. 8H), demonstrating the high biosecurity of IC@PCH NPs for in vivo application.

As illustrated in Fig. S3, mice treated with IC@PCH NPs + laser demonstrated optimal anticancer efficacy. Before treatment, a tumor was evident on the right hind limb of the mouse. By day 4 posttreatment, the tumor region began scabbing. By day 12, the wound started healing. By day 16, a solitary small scab was observed, accompanied by adjacent scar tissue formation. By day 20, the skin had nearly completed its healing process, and no recurrence of the tumor was evident. At the same time, the control and laser-only groups showed a significant increase in tumor volume, while the I@PCH NPs + laser group and the C@PCH NPs group were able to alleviate the increase in tumor volume to a certain extent compared with the control group. The D@PCH NPs + Cu^2+^@PCH NPs group and the IC@PCH NPs group also had a slight inhibitory effect on tumor growth. In conclusion, the HA-coated targeted drug delivery system, in conjunction with photothermal therapy and chemotherapy, exhibits potent synergistic antitumor activity, successfully preventing the recurrence of tumors. Also, the favorable recovery of the mice at the skin wound provides confidence in applying this treatment strategy to clinical breast-conserving therapy.

On the second day after treatment, one mouse from each group was randomly selected to be executed and used for immunohistochemical staining and immunofluorescence staining (Fig. 8I). H&E staining showed that tumor cells in the control and laser-only groups were blue-stained, with a large proportion of nucleoplasm and nuclear fragmentation was visible. Some tumor cells in the D@PCH NPs + Cu^2+^@PCH NPs group and the IC@PCH NPs group showed nuclear consolidation or fragmentation. Extensive nuclear consolidation of the tumor cells was seen in the I@PCH NPs + laser group, and extensive nuclear fragmentation of the tumor cells was seen in the C@PCH NPs group. Most of the tumor cells had nuclear fragmentation, and extensive nuclear lysis was seen in the IC@PCH NPs + laser group. This indicates that chemotherapy + targeting treatment, photothermal + targeting treatment, and photothermal + chemotherapy + targeting treatment can all induce tumor cell necrosis, and the degree of damage is deepened sequentially. The results of the TUNEL assay and PCNA staining have been widely recognized as indicators of apoptosis and tumor proliferation, respectively. The positive index of the TUNEL assay was highest in the IC@PCH NPs + laser group, with some in the D@PCH NPs + Cu^2+^@PCH NPs group, IC@PCH NPs group, C@PCH NPs, and I@PCH NPs + laser group, and was almost invisible in the control and laser-only groups, indicating that the targeted chemo-phototherapy combination therapy effectively induced TNBC cells’ death. In addition, the PCNA staining level was the lowest in the IC@PCH NPs + laser group, suggesting that the targeted photothermal- chemotherapy significantly suppressed tumor cell proliferation. The control and laser-only groups showed low expression of TUNEL and high expression of PCNA, and the absence of a notable disparity between the 2 groups suggests that the utilization of a 808-nm laser poses no adverse effects on tissue at the molecular level. These exciting, superb results provided an experimental basis for minimally invasive treatment of TNBC.

### In vitro and in vivo multimodal imaging and in vivo distribution

Studies suggest that an ideal nanomedicine could accomplish effective tumor treatment while also providing precise imaging guidance and immediate therapeutic effect analysis, especially for highly malignant TNBC. Multimodal imaging techniques, such as combining ultrasound with computed tomography technology or fluorescence with MRI, have emerged as a recent research focus [43,44]. A specific imaging technique possesses unique advantages yet also presents inherent technical limitations. Incorporating multimodal imaging techniques on a single nanoplatform effectively addresses these shortcomings for achieving multistep-free theranostics [45,46]. Based on this, we prepared IC@PCH NPs as triple-mode contrast agents for PA/MR/fluorescence imaging, designed to integrate diagnosis and treatment.

**Fig. 9.**
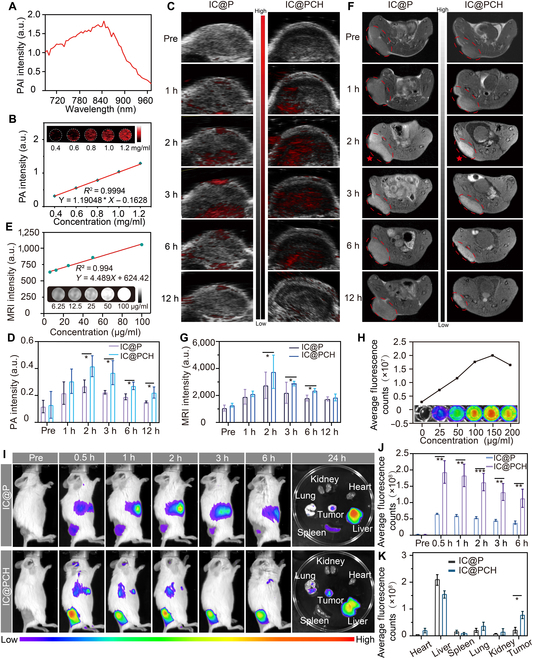
In vitro and in vivo multiple imaging. (A) The optimal excitation wavelength of IC@PCH NPs was identified by comprehensive spectral scanning. (B) Photoacoustic (PA) images (inset) and corresponding signal intensities of IC@PCH NPs at increasing concentrations. (C) PA images and (D) quantitative analysis of tumor sites at different times after intravenous injection of IC@P NPs and IC@PCH NPs; *n* = 3. (E) T1-weighted magnetic resonance (MR) images (inset) and intensities of Cu^2+^ at increased concentration. (F) T1-weighted MR images and (G) quantitative analysis of tumors in 4T1 tumor-bearing mice at various times following intravenous injection of IC@P NPs and IC@PCH NPs; *n* = 3. Nanoparticle aggregation zones are circled in dotted lines, and peak MRI times are marked with red pentagrams. (H) Fluorescence images (inset) and intensities of IC@PCH NPs at increased concentration. (I) In vivo fluorescence images of mice treated with IC@P NPs and IC@PCH NPs at different time points, and ex vivo fluorescence images of key organs and tumors excised from mice at 24 h posttreatment. (J) Tumor site fluorescence intensity quantification; *n* = 3. (K) Ex vivo fluorescence intensity analysis in major organs and tumors; *n* = 3. All results are expressed as mean ± SD, with statistical significance indicated as *P < 0.05 and **P < 0.01.

PA imaging is an innovative technique based on the PA effect. The full-wavelength scanning results showed that the IC@PCH NPs had a distinct absorption peak at 845 nm in the NIR region (Fig. 9A). The optoacoustic properties of the NPs were attributed to the combined effect of ICG as well as CuET; the latter 2 optoacoustic signal waveforms are shown in Fig. S4A and B. A laser at a wavelength of 845 nm was used to excite the PA signals of the NPs, and PA images showed that the in vitro PA signals were progressively enhanced with the concentration of IC@PCH NPs in a linear manner (Fig. 9B). To noninvasively and precisely monitor the accumulation of IC@P NPs and IC@PCH NPs at tumor sites in real time and acquire tumor morphology, we injected the NPs intravenously and conducted PA imaging at the tumor site, visualizing the therapeutic process (Fig. 9C). After the injection of IC@P NPs and IC@PCH NPs, the tumor site’s PA signal was found to enhance up to its peak intensity at 2 h postinjection. ICP@CH NPs were more concentrated at the tumor site than the IC@P NPs group at each time point, demonstrating their better tumor-targeting ability (Fig. 9D). Both the ICG@PCH NPs group and CuET@PCH NPs group showed weaker fluorescence at the tumor site than the IC@PCH NPs group, again indicating that the PA imaging ability of IC@PCH NPs originated from the combined effect of ICG and CuET (Fig. S5A and B).

Compared to PA imaging, MRI has superior soft tissue contrast and infinite penetration depth. T1-weighted MR images of the IC@PCH NPs’ dispersed system in vitro showed no significant MR signal enhancement ability (Fig. S4C), whereas T1-weighted images of Cu^2+^ in vitro and IC@PCH NP solution in vivo showed MR enhancement, and there was a linear correlation between the concentration of Cu^2+^ and MR signal (Fig. 9E). This suggests that IC@PCH NPs can metabolize to Cu^2+^ at the tumor site and have desirable contrast-enhanced MRI capabilities for subsequent imaging.

Following injection of IC@P and IC@PCH NP suspension, with the gradual accumulation of NPs, the tumor demonstrates peak MRI signal strength at 2 h postinjection (Fig. 9F). HA-coated IC@PCH had a stronger tumor-targeting ability than IC@P and prolonged the NP retention time (Fig. 9G), which was consistent with PA imaging results. PA imaging can provide high-spatial-resolution microstructural images of the tumor site, reflecting the accumulation of NPs at the tumor site, and it is relatively inexpensive and portable. However, its depth of penetration is severely limited by reflection, refraction, and absorption of acoustic waves, and its whole-body imaging capability is poor [47]. MRI not only provides detailed tissue information that identifies fine (~3 mm) as well as precise anatomical information in vivo but also allows scanning of the entire body without tissue depth limitations, but it is expensive and has a long imaging time [48]. Therefore, integrating MR/PA imaging modes can effectively guide and oversee the entire treatment process and provide valuable complementary information for clinical applications.

Fluorescence imaging is also a frequently used imaging modality in clinical practice. It has the advantages of high sensitivity, high specificity, and multicolor imaging, but the limited depth of penetration of light into the tissue (micrometers to centimeters, depending on the wavelength) [49]. Thus, fluorescence imaging can be used as a complement to the 2 imaging modalities mentioned above [50]. We investigated the ex vivo and in vivo fluorescence imaging ability and biodistribution of IC@P NPs and IC@PCH NPs. The fluorescence intensity of IC@PCH NPs showed a linear enhancement relationship at concentrations less than 150 μg/ml (Fig. 9H). Notably, the maximum fluorescence intensity of tumor tissue was observed after 0.5 h of accumulation of IC@P NPs and IC@PCH NPs (Fig. 9I). The peak time was about 1.5 h earlier than that of PA imaging and MRI modalities, which may be mainly due to the short circulating half-life, high safety index, and rapid hepatic clearance of ICG. Quantitative analysis of fluorescence intensity at the tumor site similarly demonstrated that HA promotes the accumulation of IC@PCH NPs at the tumor site (Fig. 9J).

To observe the biodistribution of IC@P, IC@PCH NPs, major organs (including the heart, liver, spleen, lungs, and kidneys) and tumors were collected for ex vivo fluorescence imaging at the end of the whole observation period (Fig. 9I). In ex vivo bioimaging, the fluorescence signal of IC@PCH NPs within the tumor increased about 2.79-fold compared with that of IC@P NPs, demonstrating that the fluorescence imaging ability was greatly improved, which was attributed to its good targeting ability as well as the increase in ICG loading rate (Fig. 9K).

Based on these findings, we conclude that each component of the triple-modality probe enhances the other modalities, ultimately providing more accurate information in biological systems. The superior tumor accumulation efficiency of IC@PCH NPs was attributed to the specific binding of CD44 overexpressed on tumor cell membranes to HA. Therefore, HA-modified IC@PCH NPs may offer highly sensitive and specific outcomes for nanomedicine TNBC monitoring.

## Conclusion

In conclusion, we have successfully constructed CD44-receptor-targeted IC@PCH NPs and proposed a breast-conserving therapeutic strategy combining chemotherapy and photothermal therapy for a population of young women with TNBC. NPs bind to tumor cells with high CD44 receptor expression through a HA shell and release the drug in an acidic TME. The IC@PCH NPs were effective in killing tumors by photothermal-chemotherapy, leading to pyroptosis. Pyroptosis breaks through the blockade of TNBC to conventional therapeutic modalities, finding new therapeutic targets and re-sensitizing TNBC to treatment. The repurposing of the old drug DSF overcomes the insensitivity of TNBC while reducing the drug development cycle, offering new possibilities for curing TNBC. In addition, copper ions mediate the generation of PA and MRI signals, while ICG mediates the generation of PA and FI signals, which together detect and guide the therapeutic process, better suggesting occult tumorigenic lesions. These findings present new insights into DSF–copper NP-based targeted delivery systems for pyroptosis-induced augmentation of photothermal-chemotherapy against TNBC. Given the Food and Drug Administration approval of all components within our strategy, coupled with the considerable attention garnered by combination therapy, our research holds potential for advancing toward clinical implementation.

## Data Availability

All data generated or analyzed during this study are included in this published article.
