# Supplementary material for: Breaking the Chains of Therapeutic Blockade: Pyroptosis-Induced Photothermal-Chemotherapy with Targeted Nanoprobes in Triple-Negative Breast Cancer
Source: Biomater Res. 2025 May 15;29:0200. doi: 10.34133/bmr.0200 (PMC12079191; doi:10.34133/bmr.0200)
Supplement: Supplementary 1 — Figs. S1 to S5 Table S1 [file bmr.0200.f1.docx]

**Supporting Information**

**Breaking the Chains of Therapeutic Blockade: Pyroptosis-Induced Photothermal-Chemotherapy with Targeted Nanoprobes in Triple-Negative Breast Cancer**

Zuying Li^1,2†^, Kexiao Yu^3†^, Youde Cao^4,5^, Hui Yuan^4,5^, Lingcheng Wu^2,4^, Linyan Xiong^4,5^, Yi Tang^1*^, Bing Liang^4,5*^

1 Department of Ultrasound of Children’s Hospital of Chongqing Medical University, National

Clinical Research Center for Child Health and Disorders, Ministry of Education Key Laboratory of

Child Development and Disorders, Chongqing Engineering Research Center of Stem Cell Therapy,

Children’s Hospital of Chongqing Medical University, Chongqing 400014, P.R. China.

2 Department of Ultrasound & Chongqing Key Laboratory of Ultrasound Molecular Imaging, The

Second Affiliated Hospital of Chongqing Medical University, Chongqing 400010, P. R. China.

3 Department of Orthopedics, Chongqing Traditional Chinese Medicine Hospital, The First

Affiliated Hospital of Chongqing College of Traditional University of Chinese Medicine,

Chongqing 400021, P. R. China.

4 Department of Pathology from College of Basic Medicine, and Molecular Medicine Diagnostic & Testing Center, and Department of Clinical Pathology Laboratory of Pathology Diagnostic Center,

Chongqing Medical University, Chongqing 400016, P. R. China.

5 Department of Pathology, The First Affiliated Hospital of Chongqing Medical University,

Chongqing, 400042, P.R. China.

† These authors contributed equally to this work as co-first authors.





**Table S1.** Size distribution of IC@P NPs、IC@PC NPs、IC@PCH NPs





**Fig. S1.** (A) UV-visible and infrared absorbance spectra of CuET solution with various concentrations. (B) Standard curve of CuET solution. (C) UV-visible and infrared absorbance spectra of ICG solution with various concentrations. (D) Standard curve of ICG solution.





**Fig. S2.** Stability and drug release of IC@PCH NPs. (A)The size distribution, ζ potential, and digital photos of the IC@PCH NPs dispersed in PBS within 7 days. (B)The kinetics of CuET release from IC@PCH NPs under various pH conditions.


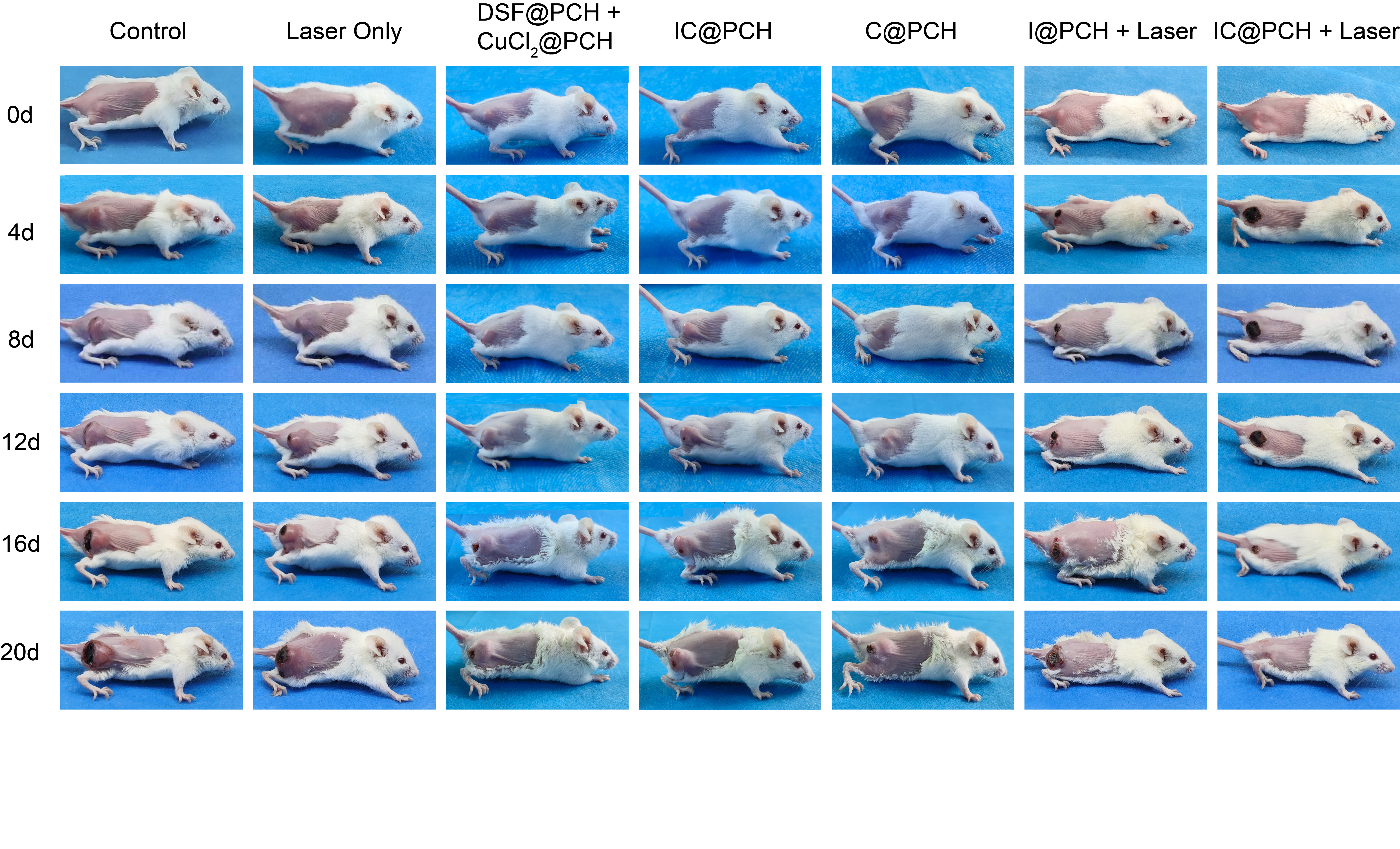


**Fig. S3.** Representative digital pictures of 4T1 tumor-bearing mice during 20 days period after intravenous administration of different formulations.


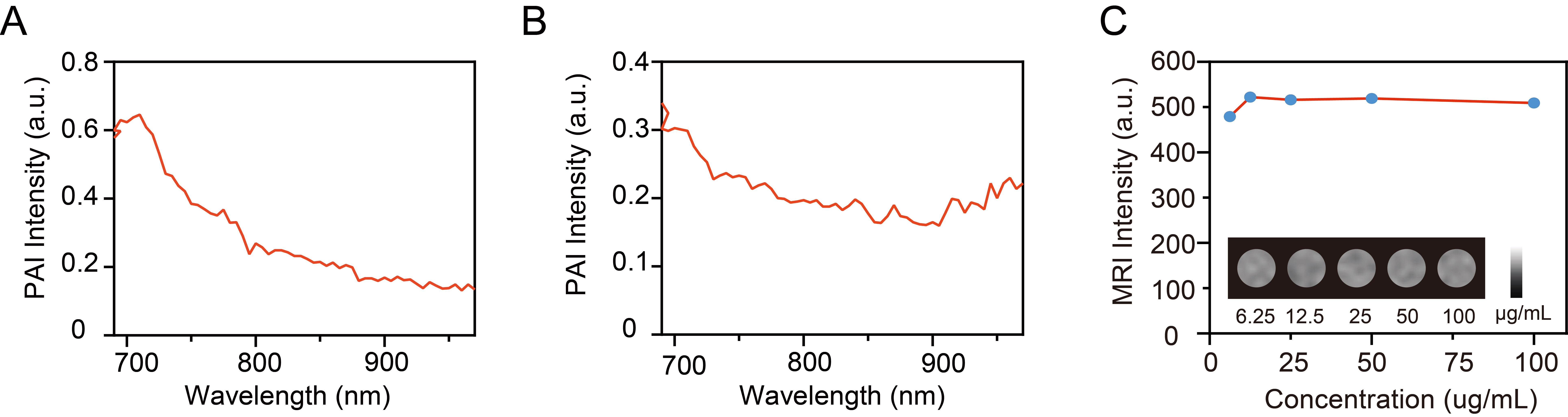


**Fig. S4.** The detection of optimal excitation wavelength of (A) ICG and (B) CuET. (C)T1weighted MR images (inset) of IC@PCH NPs at different concentrations at increased concentrations from the corresponding signal intensities.


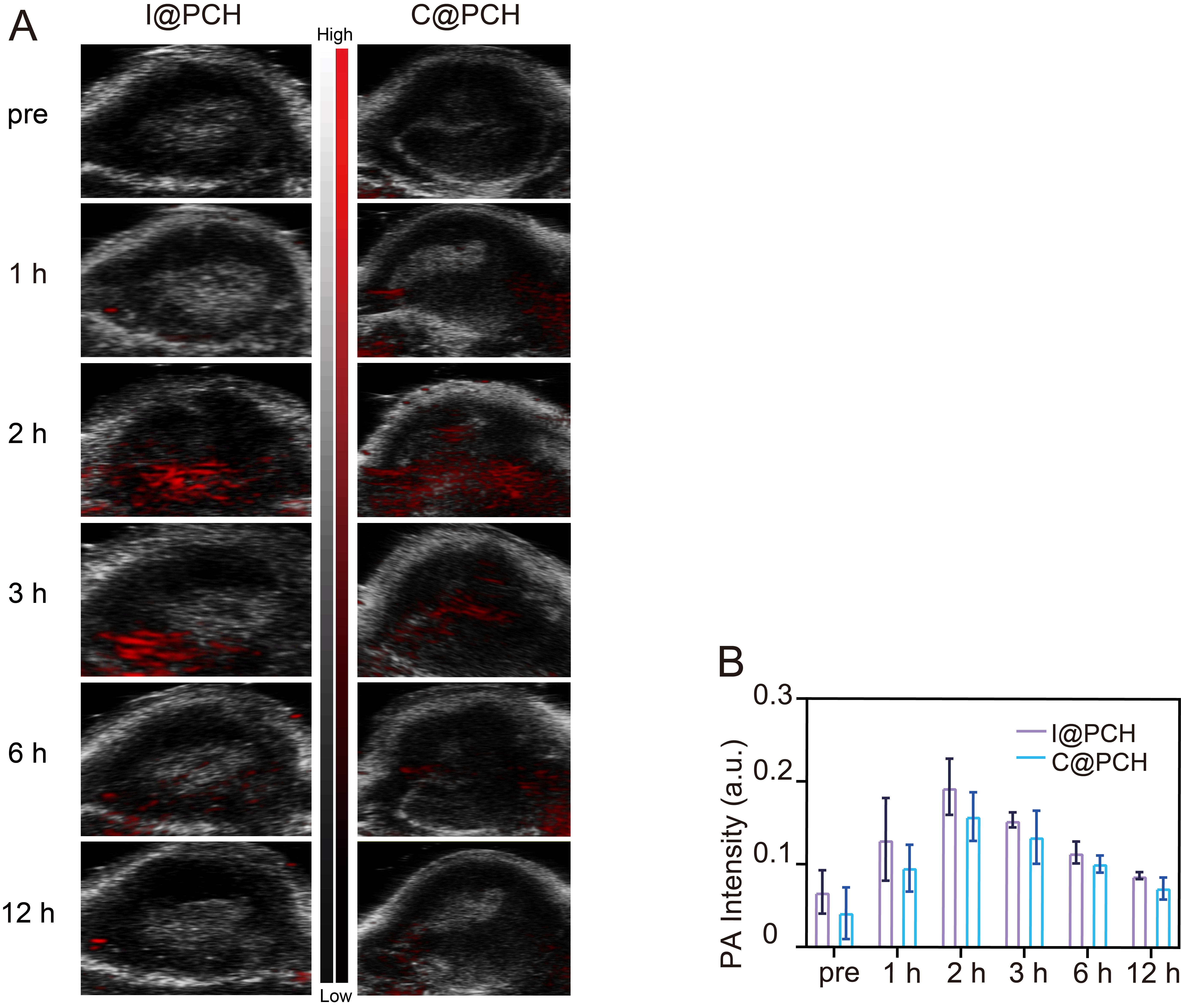


**Fig. S5.** (A) PA images and (B) Quantitative analysis of tumor sites at different times after intravenous injection of I@PCH NPs and C@PCH NPs; n = 3.
